# Supplementary material for: Crop switching reduces agricultural losses from climate change in the United States by half under RCP 8.5
Source: Nat Commun. 2020 Oct 5;11:4991. doi: 10.1038/s41467-020-18725-w (PMC7536391; doi:10.1038/s41467-020-18725-w)
Supplement: Supplementary file 3 — Reporting Summary [file 41467_2020_18725_MOESM3_ESM.pdf]

nature research

Corresponding author(s): James Rising

Last updated by author(s): Aug 4, 2020

Reporting Summary

Nature Research wishes to improve the reproducibility of the work that we publish. This form provides structure for consistency and transparency in reporting. For further information on Nature Research policies, see our Editorial Policies and the Editorial Policy Checklist.

Statistics

For all statistical analyses, confirm that the following items are present in the figure legend, table legend, main text, or Methods section.

n/a	Confirmed	
☐	☒	The exact sample size (*n*) for each experimental group/condition, given as a discrete number and unit of measurement
☒	☐	A statement on whether measurements were taken from distinct samples or whether the same sample was measured repeatedly
☐	☒	The statistical test(s) used AND whether they are one- or two-sided<br>*Only common tests should be described solely by name; describe more complex techniques in the Methods section.*
☐	☒	A description of all covariates tested
☐	☒	A description of any assumptions or corrections, such as tests of normality and adjustment for multiple comparisons
☐	☒	A full description of the statistical parameters including central tendency (e.g. means) or other basic estimates (e.g. regression coefficient) AND variation (e.g. standard deviation) or associated estimates of uncertainty (e.g. confidence intervals)
☐	☒	For null hypothesis testing, the test statistic (e.g. *F*, *t*, *r*) with confidence intervals, effect sizes, degrees of freedom and *P* value noted<br>*Give P values as exact values whenever suitable.*
☐	☒	For Bayesian analysis, information on the choice of priors and Markov chain Monte Carlo settings
☐	☒	For hierarchical and complex designs, identification of the appropriate level for tests and full reporting of outcomes
☒	☐	Estimates of effect sizes (e.g. Cohen's *d*, Pearson's *r*), indicating how they were calculated

Our web collection on statistics for biologists contains articles on many of the points above.

Software and code

Policy information about availability of computer code

Data collection	No software was used to collect data.
Data analysis	All model and display code is available in a Zenodo code repository, DOI 10.5281/zenodo.3909637. The optimization model is constructed using the landuse component of AWASH 2.0, available at https://github.com/AmericasWater/awash. The Bayesian model uses JAGS 4.3.0 and the optimization model uses Gurobi Optimizer 9.0.2.

For manuscripts utilizing custom algorithms or software that are central to the research but not yet described in published literature, software must be made available to editors and reviewers. We strongly encourage code deposition in a community repository (e.g. GitHub). See the Nature Research guidelines for submitting code & software for further information.

Data

Policy information about availability of data

All manuscripts must include a data availability statement. This statement should provide the following information, where applicable:

- Accession codes, unique identifiers, or web links for publicly available datasets
- A list of figures that have associated raw data
- A description of any restrictions on data availability

The data used in making the charts and tables in this paper is available at DOI 10.5281/zenodo.3889144.

Field-specific reporting

Please select the one below that is the best fit for your research. If you are not sure, read the appropriate sections before making your selection.

- [ ] Life sciences
- [ ] Behavioural & social sciences
- [x] Ecological, evolutionary & environmental sciences

For a reference copy of the document with all sections, see nature.com/documents/nr-reporting-summary-flat.pdf

Ecological, evolutionary & environmental sciences study design

All studies must disclose on these points even when the disclosure is negative.

Study description	The effect of changing weather on yields is determined, and this relationship is used to study the potential for climate change adaptation.
Research sample	Yields for six crops (barley, corn, cotton, rice, soybeans, and combined winter and spring wheat) across the United States at a county level, from 1949 - 2009.
Sampling strategy	All available data was used.
Data collection	J.R and N.D. collected from the USDA Quick Stats tool by selecting the desired crops, years, and all counties.
Timing and spatial scale	From 1949 to 2009, across the continental United States at a county level.
Data exclusions	All available data was used.
Reproducibility	Analysis results derive from the available data. The Bayesian model was run multiple times during the research, each time producing nearly identical (<2% change) results.
Randomization	Unobserved covariates were controlled for using fixed effects, which assumes they produce a constant multiplicative effect, and hierarchical latent variables, which assumes that parameters vary of space but are constant in time.
Blinding	The structure of the model was determined prior to its fitting and the production of results. All observations were considered "treated", so no blinding for group allocation was possible.

Did the study involve field work? [ ] Yes [x] No

Reporting for specific materials, systems and methods

We require information from authors about some types of materials, experimental systems and methods used in many studies. Here, indicate whether each material, system or method listed is relevant to your study. If you are not sure if a list item applies to your research, read the appropriate section before selecting a response.

Materials & experimental systems

n/a	Involved in the study	
[x]	[ ]	Antibodies
[x]	[ ]	Eukaryotic cell lines
[x]	[ ]	Palaeontology and archaeology
[x]	[ ]	Animals and other organisms
[x]	[ ]	Human research participants
[x]	[ ]	Clinical data
[x]	[ ]	Dual use research of concern

Methods

n/a	Involved in the study	
[x]	[ ]	ChIP-seq
[x]	[ ]	Flow cytometry
[x]	[ ]	MRI-based neuroimaging
