# Supplementary material for: Crop switching reduces agricultural losses from climate change in the United States by half under RCP 8.5
Source: Nat Commun. 2020 Oct 5;11:4991. doi: 10.1038/s41467-020-18725-w (PMC7536391; doi:10.1038/s41467-020-18725-w)
Supplement: Supplementary file 1 — Supplementary Information [file 41467_2020_18725_MOESM1_ESM.pdf]

SUPPLEMENTARY INFORMATION

Crop switching reduces agricultural losses from climate change in the United States by half under RCP 8.5

Rising and Devineni

Supplementary Material

Crop	Observations	Counties	Year.Span
Barley	52 676 county-years	2111	1949 - 2009
Corn	131 842 county-years	2815	1949 - 2009
Cotton	33 333 county-years	980	1949 - 2009
Rice	5684 county-years	147	1949 - 2009
Soybeans	91 170 county-years	2222	1949 - 2009
Wheat	120 289 county-years	2821	1949 - 2009

Supplementary Table 1. Yield source data. Observation counts and county and year coverage for the crop yield data.

Supplementary Note 1: Crop Water Deficit Index Calculations

The Crop Water De cit Index (CDI) used here computes the maximum cumulative de cit over a growing season between a crop's daily water requirement for optimal growth and the daily rainfall. Given an n-year record of daily data, CDI calculates the day-by-day accumulation of de cit in water supply in each of the n growing seasons. The maximum of these daily de cit values within each season is then taken to be the value of the index for that season. This gives a worst-case scenario in terms of the seasonal water stress on the crop, and can therefore be interpreted as the amount of water that should be drawn from external storage to meet water demand [Ravindranath et al., 2018, Devineni et al., 2013, 2015, Etienne et al., 2016, Ho et al., 2016].

De cit is estimated as the di erence between the seasonal crop water requirement and renewable water supply for each crop, in a given county during the growing season. We assume that the rainfall for each county is available as the renewable water supply, which is given as

(1) $$S_{jdt} = \quad P_{jdt}$$

In equation 1, P_{jdt} is the rainfall in a given county j, for any given day d, for the year t. is the parameter that determines the fraction of rainfall that can be utilized by the crops in a location. In this study, we used the distribution of from Devineni et al. [2015]. These are estimated based on the long-term runo ratio of each county. We estimate the water use for a given crop based on the expected growth stage and daily evapotranspiration as

(2) $$D_{jdt} = k_{cd}\,(\mathrm{ET}_0)_{jdt}$$

In equation 2, k_{cd} is the FAO recommended crop coe cient for crop c. It is the ratio of actual evapotranspiration (ET_a) of a given crop under non-stressed conditions to reference crop evaporation (ET_0). It represents crop-speci c water use at various growth stages of the crop and is typically derived empirically based on local climatic conditions [Doorenbos and Pruitt, 1977]. The accumulated de cit over a season is then given as

(3) $$\text{de cit}_{jdt} = \max(\text{de cit}_{j;d\ \ 1;t} + D_{jdt} \quad S_{jdt}; 0) \qquad \text{where de cit}_{j;d=0;t} = 0$$

(4) $$\mathrm{CDI}_{j;t} = \max_{d}(\text{de cit}_{jdt}) \qquad \text{where } t = 1 \quad n$$

de cit$_{jdt}$ refers to the accumulated daily de cit, D_{jdt} to the total daily crop water demand, S_{jdt} to the total daily water supply for geographical location j, and day d, in year t; t refers to a calendar or cropping year; and n is the total number of years in the analysis.

The CDI index is an improvement on the metrics derived using monthly or seasonal rainfall and crop demand because it focuses on the rainfall distribution within the season relative to the crop water demand. CDI therefore accounts for the timing of planting, di erent stages of crop growth, and the timing and distribution of rainfall in the season. Thus, it can discriminate between two years which have the same total rainfall, but di er in that one may have rainfall distributed uniformly over the season through modest rainfall events, while the other may have a few intense rain events separated by long dry periods. We are using the following datasets to estimate the water supply, crop water demand and crop water de cit for each county:

(1) Gridded rainfall data from 1949 to 2010 (62 years) available at $1/8$ spatial resolution developed by Maurer et al. [2002] were aggregated to 3111 counties and used to estimate the water supply in each county.

(2) Daily air temperature and wind speed from Maurer et al. [2002] available at the same spatial and temporal resolution were used to estimate the potential evapotranspiration for each grid (and then aggregated to county level) based on the Penman method [Zotarelli et al., 2010]. The FAO Penman method is recommended as the standard method for estimating evapotranspiration [Allen et al., 1998].

Supplementary Note 2: Growing Degree-Days and Extreme Degree-Days Calculations

The growing degree-days (GDDs) and extreme degree-days (EDDs) are estimated according to the methods in Schlenker and Roberts [2009]. We use daily temperature data from 1949 - 2010 from She eld et al. [2006], available at $1/8$ resolution over the United States. Minimum and maximum daily temperatures are averaged over each county, and the temperature range within each day is approximated by a sinusoid between these temperatures. The growing degree-day contribution for each day is calculated as the integral of the portion of sinusoid between a lower and upper threshold, and extreme degree-days are the integral above the upper threshold [Snyder, 1985]. The temperature thresholds are listed in Supplementary Table 2, and are derived from literature and DSSAT crop models.

We calculate GDDs and EDDs over the crop calendars from Sacks et al. [2010], extended to areas where the crops are not currently grown, and applying the mean planting and harvesting dates by county. In the case of wheat, we use the crop calendar for spring wheat in counties where it is the dominant variety, and otherwise use winter wheat.

Crop	GDD Range	EDD Range	Notes
Barley	0 C to 15 C	above 15 C	From DSSAT's `BarleyGDD.CRO`
Corn	8 C to 29 C	above 29 C	From Schlenker and Roberts [2009]
Cotton	12 C to 31 C	above 31 C	From DSSAT's `CottonGDD.CRO` ^{y}
Soybeans	5 C to 29 C	above 29 C	From DSSAT's `SoybeanGDD.CRO` ^{z}
Rice	8 C to 29 C	above 29 C	From DSSAT's `PaddyRiceGDD.CRO` ^{z}
Wheat	0 C to 26 C	above 26 C	From DSSAT's `WheatGDD.CRO`

Supplementary Table 2. Growing degree-day calculation parameters by crop. Where parameters are taken from a DSSAT GDD model, the lower bound for GDDs is taken from the model, but the upper bound is adjusted according to the table notes since the GDD-EDD calculation still produces yield increases for some temperatures above the GDD upper bound. The temperature ranges for corn were estimated by a parameter sweep by Schlenker and Roberts [2009]. These have fairly low upper bounds, so the GDD upper bound and EDD lower bound were taken as given. ^{y} Cotton had a high upper bound and so was replaced with 31 C. ^{z} The upper bound for GDDs was taken as 1 degree less than given by the DSSAT model.

Supplementary Note 3: Multilevel Bayesian Model Details

A hierarchical Bayesian approach was used to model yields within and across counties. Our goal was to estimate the distribution of crop yields in each of the 3111 counties in the United States. The time series (1949 - 2009) of the crop yield (for each crop) in each county is informed by weather predictors (crop speci c water de cit index (CDI), growing degree days (GDD), extreme degree days (EDD)) and technology improvements (using a time trend). In addition, we have bioclimatic attributes [Booth et al., 2014] and irrigation measures [Portmann et al., 2010], which are constant over time, averaged over each county, as county level covariates.

Our motivation for a hierarchical Bayesian approach is that we wish to allow both variation in how yields respond to weather predictors across counties where data is available, but also be able to predict the general determinants of these variations for counties that are not observed with a given crop. To explain this more clearly, consider the e ect of a single weather predictor (say, GDDs) on crop yield in each county. If the true response is exactly the same for all counties, using a single coe cient for all counties and pooling all the data into the same regression would be e ective since that would reduce the uncertainty associated with parameter estimation. However, the response across the counties may vary systematically due to local conditions or idiosyncratically. The hierarchical model allows for both systematic and idiosyncratic variation in the coe cients.

The hierarchical model consists of two levels. The individual regression coe cients for each county on each climate predictor are represented at the rst level. The errors at this level represent the within county variations that can be attributed to reporting errors and residual variability beyond what is explained by weather predictors. The second level models how these regression coe cients vary across counties using local bioclimate attributes such as mean annual temperature and its seasonality. We also use fraction of the crop under irrigation, to capture how crop responsiveness changes to irrigation. The errors at this level represent variations in the climate response between

counties that cannot be explained by county level covariates. We fit a hierarchical regression model to the crop yield data in all counties simultaneously while accounting for systematic unexplained variance among the 3111 counties. The modeling equations follow.

The crop yield $\mathbf{Y}$ (the county time series of the crop yield were obtained from the USDA National Agriculture Statistics Service, NASS [USDA NASS, 2018]) are assumed to come from a probability density function f($\mathbf{Y} \mid \boldsymbol{\theta}$), where $\boldsymbol{\theta}$ is the parameter vector. In the current application, we consider that log($\mathbf{Y}$) is normally distributed. The first level of the model considers that in each county i and year t, log(Y_{it}) is described by a Normal distribution with time varying mean $\ _{it}$ that is a linear combination of the four chosen predictors with county-specific coefficients $\ _i^k$ (k indexes the predictors) and intercept $\ _i$. The second level of the model considers that the intercepts $\boldsymbol{\alpha}$ and the regression coefficient matrix $\ ^k$ can be estimated using county level covariates. The errors from the regression models are considered to be independent and identically-distributed with a 0 mean and a variance that is estimated as part of the model.

(5)

$$\mathbf{Level\ 1}: \log(Y_{it}) \sim N(\ _{it};\ ^2_i)$$
$$_{it} = \ _i + \ ^1_i t + \ ^2_i \mathrm{CDI}_{it} + \ ^3_i \mathrm{GDD}_{it} + \ ^4_i \mathrm{EDD}_{it}$$
$$\mathbf{Level\ 2}: \ _i \sim N(\ _i;\ ^2)$$
$$_i = a_0 + \sum_{j=1}^{\#covar} b^0_j \mathrm{covar}_{ij}$$
$$^k_i \sim N(\ ^k_i;\ ^2_k) \quad \text{for } k = 1 ::: 4$$
$$^k_i = a_k + \sum_{j=1}^{\#covar} b^k_j \mathrm{covar}_{ij} \quad \text{for } k = 1 ::: 4$$
$$\mathbf{Priors}: \ ^2_i \quad U(0; 100) \quad \text{for } i = 1 ::: 3111$$
$$^2 \quad U(0; 100)$$
$$^2_k \quad U(0; 100) \quad \text{for } k = 1 ::: 4$$
$$a_0 \quad N(0; 100)$$
$$a_k \quad N(0; 100) \quad \text{for } k = 1 ::: 4$$
$$b_0 \quad N(0; 100)$$
$$b^k_j \quad N(0; 100) \quad \text{for } k = 1 ::: 4; j = 1 ::: 19$$

We assumed a wide uniform prior distribution for the variance terms and uninformative normal priors for the coefficients of the second level [Gelman, 2006]. The covariates included are annual mean temperature, isothermality (diurnal range divided by annual temperature range), temperature seasonality (standard deviation over months), annual precipitation, precipitation seasonality (coefficient of variation across months), all from Booth et al. [2014], and irrigation fraction by crop, from Portmann et al. [2010].

The joint posterior distribution p($\boldsymbol{\theta} \mid$data), of the complete parameter vector, is derived by combining the prior distributions and the likelihood functions. The joint posterior distribution of the complete parameter vector $P(\ \mid \mathbf{Y})$ is defined as follows:

$$p(\ \mid \mathbf{Y}) \propto \prod_{i=1}^{3111} \prod_{t=1}^{61} N[Y_{it} \mid \ _i + \ ^1_i t + \ ^2_i \mathrm{CDI}_{it} + \ ^3_i \mathrm{GDD}_{it} + \ ^4_i \mathrm{EDD}_{it};\ ^2_i] U[\ ^2_i \mid 0; 100]$$
$$N[\ _i \mid a_0 + \mathbf{b^0}\mathrm{covar}_\mathbf{i};\ ^2] U[\ ^2 \mid 0; 100] N[a_0 \mid 0; 100] N[\mathbf{b_0} \mid 0; 100]$$
$$N[\ ^1_i \mid a_1 + \mathbf{b^1}\mathrm{covar}_\mathbf{i};\ ^2_1] U[\ ^2_1 \mid 0; 100] N[a_1 \mid 0; 100] N[\mathbf{b_1} \mid 0; 100]$$
$$N[\ ^2_i \mid a_2 + \mathbf{b^2}\mathrm{covar}_\mathbf{i};\ ^2_2] U[\ ^2_2 \mid 0; 100] N[a_2 \mid 0; 100] N[\mathbf{b_2} \mid 0; 100]$$
$$N[\ ^3_i \mid a_3 + \mathbf{b^3}\mathrm{covar}_\mathbf{i};\ ^2_3] U[\ ^2_3 \mid 0; 100] N[a_3 \mid 0; 100] N[\mathbf{b_3} \mid 0; 100]$$
$$N[\ ^4_i \mid a_4 + \mathbf{b^4}\mathrm{covar}_\mathbf{i};\ ^2_4] U[\ ^2_4 \mid 0; 100] N[a_4 \mid 0; 100] N[\mathbf{b_4} \mid 0; 100]$$

(6)

The parameters are estimated using JAGS [Plummer, 2003, Denwood, 2016] which employs the Gibbs sampler, a Markov Chain Monte Carlo (MCMC) method for simulating the posterior probability distribution of the parameters.

Multilevel Bayesian models have become quite common as research tools for Computational Statistics. They appear for applications in causal inference, prediction, comparison, and data description, especially for multivariable problems where the investigator needs to learn something about the group as well as individual dynamics. In many cases, they directly generalize traditional regression approaches in this setting. We were interested in exploring how a structured approach to crop yield prediction using climate and geographic covariates could be approached. In general, this is a high dimensional problem that offers some interesting opportunities. In this initial effort, we took a subset of the problem and explored specifically, how pooling information based on geographic covariates could work in better estimating the crop yields and in providing equations for estimating them in regions where the crops are currently not being cultivated. The restricted goal was to consider the use of pre-calculated climate and geographic covariates as the primary predictors. We did not consider modeling uncertainty in the predictors themselves. Further, given the high-dimentionality of the problem, beyond ensuring that the coefficients in Level 1 are modeled using spatial covariates in Level 2, we did not explicitly model the spatial auto-correlation in the residuals. We also note that our model requires assumptions as to the parametric probability density models. While we verified our assumptions using traditional goodness of fit tests, the resulting model can be logically quite complex if these simple assumptions do not hold, for example, if the variables of interest are represented by heterogeneous mixtures.

Crop	Model	Mean Rhat	Mean Ne	DIC
Barley	Full dataset	1.0	3366	-7941
	Full dataset - Constant variance	1.0	2912	-4490
	Cross-validation	1.0	1468	-5494
	Cross-validation - Constant variance	1.1	1354	-4224
Corn	Full dataset	1.0	3545	-16081
	Full dataset - Constant variance	1.0	2735	42122
	Cross-validation	1.0	1764	-10562
	Cross-validation - Constant variance	1.1	1437	33536
Cotton	Full dataset	1.0	2832	20878
	Full dataset - Constant variance	1.0	2341	21936
	Cross-validation	1.0	2639	18271
	Cross-validation - Constant variance	1.0	2237	19091
Rice	Full dataset	1.0	1987	-7242
	Full dataset - Constant variance	1.0	1526	-6980
	Cross-validation	1.0	1871	-4937
	Cross-validation - Constant variance	1.0	1112	-4930
Soybean	Full dataset	1.0	2997	-55219
	Full dataset - Constant variance	1.0	2875	-44921
	Cross-validation	1.0	3104	-42757
	Cross-validation - Constant variance	1.0	3272	-36042
Wheat	Full dataset	1.0	1947	-29157
	Full dataset - Constant variance	1.0	2029	-15281
	Cross-validation	1.0	3999	-24710
	Cross-validation - Constant variance	1.0	4059	-14843

Supplementary Table 3. Convergence statistics for the full and cross-validation fits of the Bayesian model. We simulated six chains started from random initial values for the parameters to verify the convergence of the posterior distribution based on the shrink factor (Rhat) suggested by Gelman and Rubin (1992). The shrink factor compares the variance in the sampled parameters within the chains and across the chains to describe the improvement in the estimates for an increasing number of iterations. Gelman and Rubin (1992) suggest running the chains until the estimated shrink factors are less than 1.1 for all the parameters. For our application, each chain was run for a 1000 cycle burn-in to discard the initial state, followed by 4000 iterations in the adaptation phase and 250,000 samples of model parameters. The mean Rhat value for each of the models, across all parameters and counties, is shown in the table. We find a relatively low shrink factor. The table also presents the mean effective sample size in the model across all counties, and the Deviance Information Criteria (DIC), a measure of the model's predictive skill. Smaller values of the DIC indicate a better estimated predictive capacity of the model.

Supplementary Note 4: Bayesian Model Posterior Predictive Checks

Following Gelman et al. [2013], we computed the Bayesian p-values for two test-quantities - the 10th percentile of the data ($y_{[10]}$) and the range between the 90th percentile and the 10th percentile of the data ($y_{[90]} - y_{[10]}$). Bayesian p-value is defined as the probability that the replicated data (y^{rep}) could be more extreme than the observed data (y) as measured by the test quantities, i.e., $Pr(T(y^{rep}; \theta) \geq T(y; \theta)|y)$. See Supplementary Table 4.

For additional confirmation, we present the posterior distributions of the crop yields for selected counties in Supplementary Figure 1. For each of the six crops, we randomly selected two counties from the regions where they are currently cultivated and compared the posterior predictions with the observed crop yields. For each crop and each county, we show a series of box plot diagrams depicting the posterior distributions of yields from 1949 - 2009 from the cross-validated model that uses 1949 - 1994 as the training set to predict yields for 1995-2009. In other words, the yields from 1995-2009 are not used in estimating the model coefficients. The posterior distributions for 1995-2009 are hence shown as grey boxes. The observed data from 1949-2009 are depicted as red color dots. We observe from these posterior predictions that the long-term trend, as well as the inter-annual variability in the crop yields, is well captured under the cross-validation mode. During some of the years where the observed crop yields are anomalously low, the model's directionality seems to indicate a below normal yield (see, for instance, the predictions of corn in Harrison, Indiana). We also found that for some counties (Rice in Merced, CA) the model predicts a continued trend for the test set, whereas the observed trend seemed to have plateaued.

We also present in Supplementary Figure 2 - 7, an additional verification of whether the model can reproduce the correlation of observed crop yields with the three climate predictors, i.e., CDI, GDD and EDD for counties that have at least 60 years of data. Three correlation coefficients based on the observed data ($R_{CDI;y}; R_{GDD;y}; R_{EDD;y}$) are computed for each county and compared to the correlation coefficients based on the replicated data ($R_{CDI;y^{rep}}; R_{GDD;y^{rep}}; R_{EDD;y^{rep}}$). We see that the replicated data from the model is also reproducing the correlations well.

Crop	Model	Mean p-value for $y_{[10]}$	Mean p-value for $y_{[90]} - y_{[10]}$
Barley	Full dataset	0.31	0.90
	Full dataset - Constant variance	0.31	0.88
	Cross-validation	0.61	0.77
	Cross-validation - Constant variance	0.57	0.83
Corn	Full dataset	0.42	0.73
	Full dataset - Constant variance	0.35	0.78
	Cross-validation	0.66	0.74
	Cross-validation - Constant variance	0.56	0.84
Cotton	Full dataset	0.44	0.78
	Full dataset - Constant variance	0.45	0.77
	Cross-validation	0.38	0.74
	Cross-validation - Constant variance	0.38	0.78
Rice	Full dataset	0.34	0.81
	Full dataset - Constant variance	0.36	0.81
	Cross-validation	0.51	0.80
	Cross-validation - Constant variance	0.53	0.84
Soybean	Full dataset	0.31	0.74
	Full dataset - Constant variance	0.29	0.75
	Cross-validation	0.65	0.57
	Cross-validation - Constant variance	0.66	0.60
Wheat	Full dataset	0.39	0.79
	Full dataset - Constant variance	0.38	0.77
	Cross-validation	0.55	0.71
	Cross-validation - Constant variance	0.56	0.72

Supplementary Table 4. The mean p-value across all the counties is presented for each of the 24 models in the table. The p-value is defined as the the probability that replicated data coult be more extreme than test data for the given metric (the 10th percentile and the range between the 90th and 10th percentile). The tail area probabilities in all the cases are within 0.05 and 0.95, indicating that the model can replicate the observed data well.

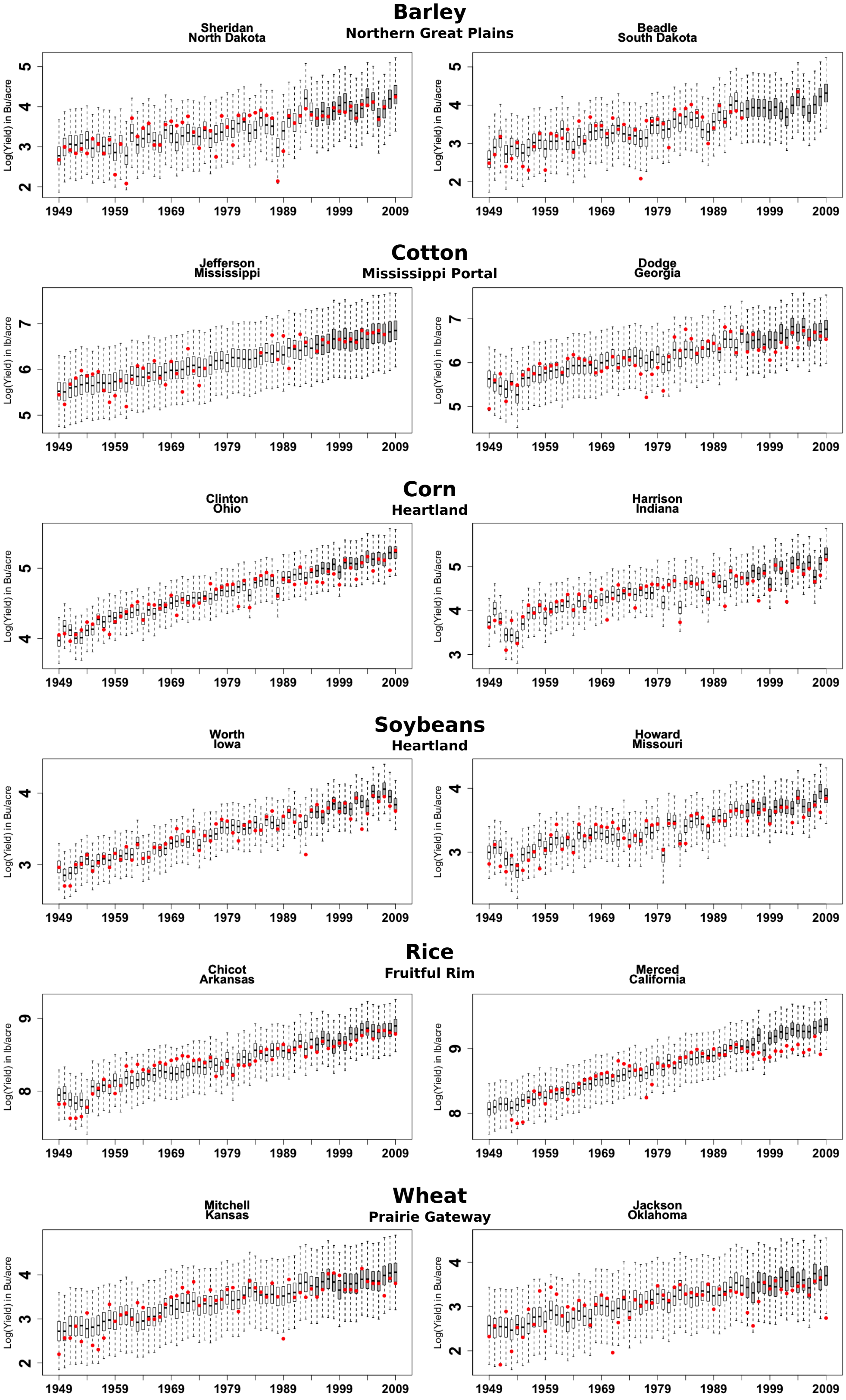

Supplementary Figure 1. Posterior distributions of the crop yields for 12 selected counties. The boxplots show 25th, 50th, and 75th percentiles of the model posterior predicted log-yields, with error bars extending to the most extreme posterior predicted value or 150% of the interquartile range, whichever is narrower. Red dots show the observed yields. Models are t to data through 1994. n=3000 posterior draws.

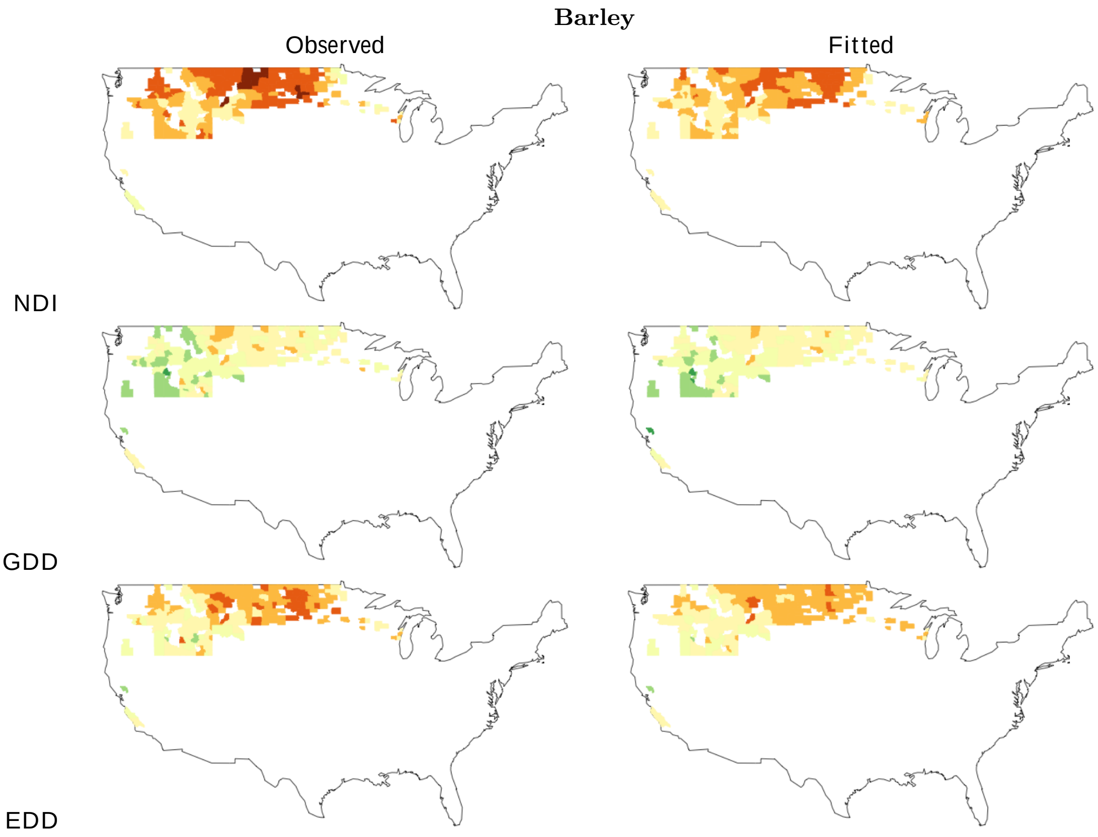

Supplementary Figure 2. Correlation coe cients between the three climate predictors and the observed Barley yield data ($R_{CDI;y}; R_{GDD;y}; R_{EDD;y}$) (left panel). Correlation coe cients between the three climate predictors and the replicated Barley yield data ($R_{CDI;y^{rep}}; R_{GDD;y^{rep}}; R_{EDD;y^{rep}}$) (right panel).

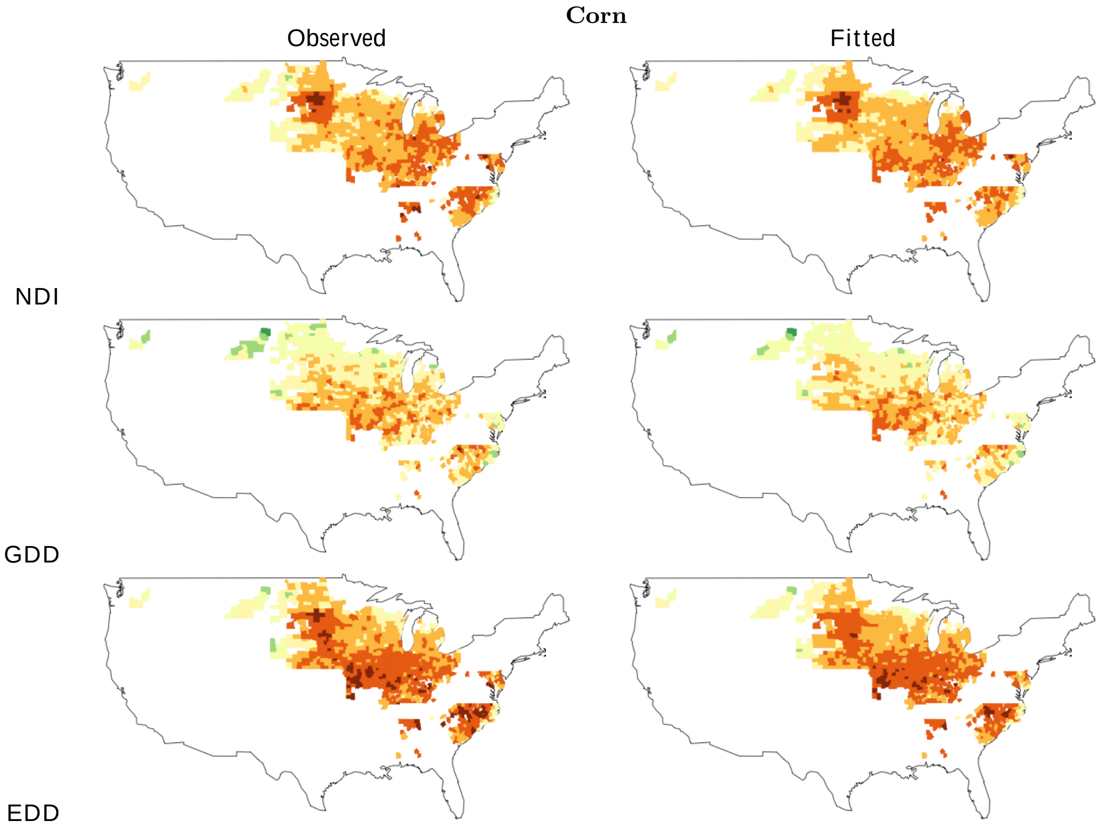

Supplementary Figure 3. Correlation coe cients between the three climate predictors and the observed Corn yield data ($R_{CDI;y}; R_{GDD;y}; R_{EDD;y}$) (left panel). Correlation coe cients between the three climate predictors and the replicated Corn yield data ($R_{CDI;y^{rep}}; R_{GDD;y^{rep}}; R_{EDD;y^{rep}}$) (right panel).

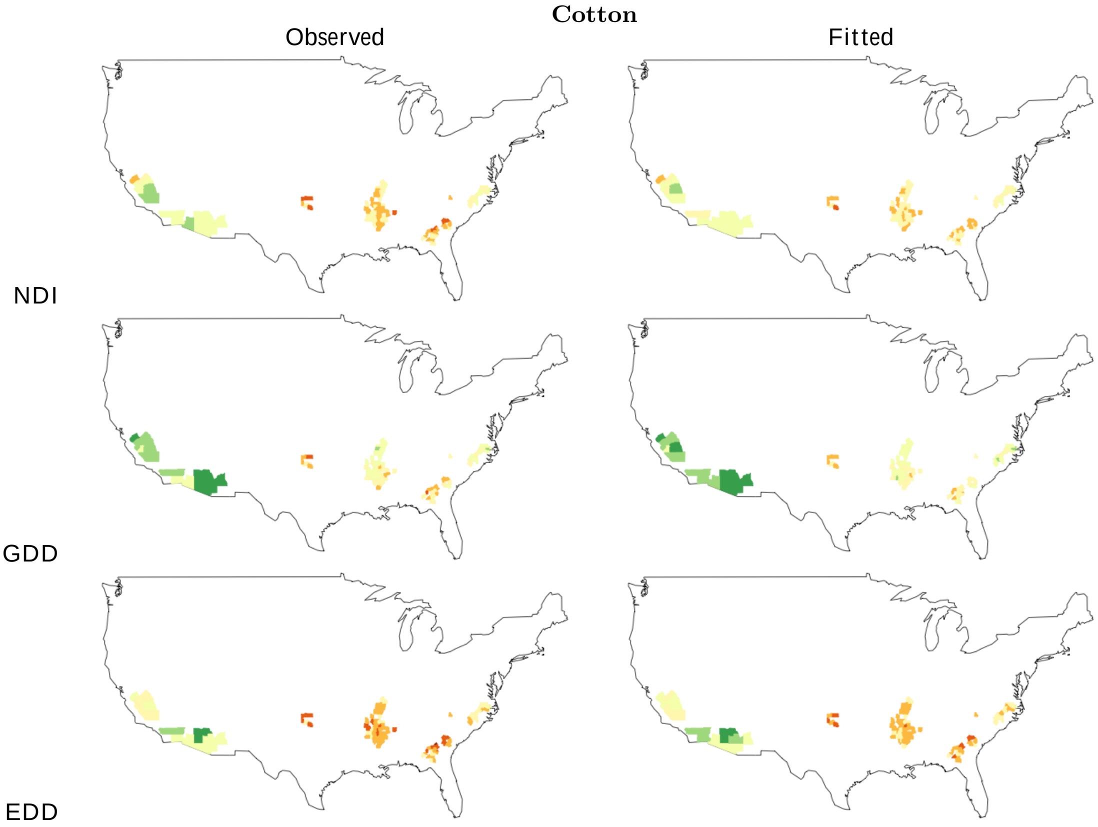

Supplementary Figure 4. Correlation coe cients between the three climate predictors and the observed Cotton yield data $(R_{CDI;y}; R_{GDD;y}; R_{EDD;y})$ (left panel). Correlation coe cients between the three climate predictors and the replicated Cotton yield data $(R_{CDI;y^{rep}}; R_{GDD;y^{rep}}; R_{EDD;y^{rep}})$ (right panel).

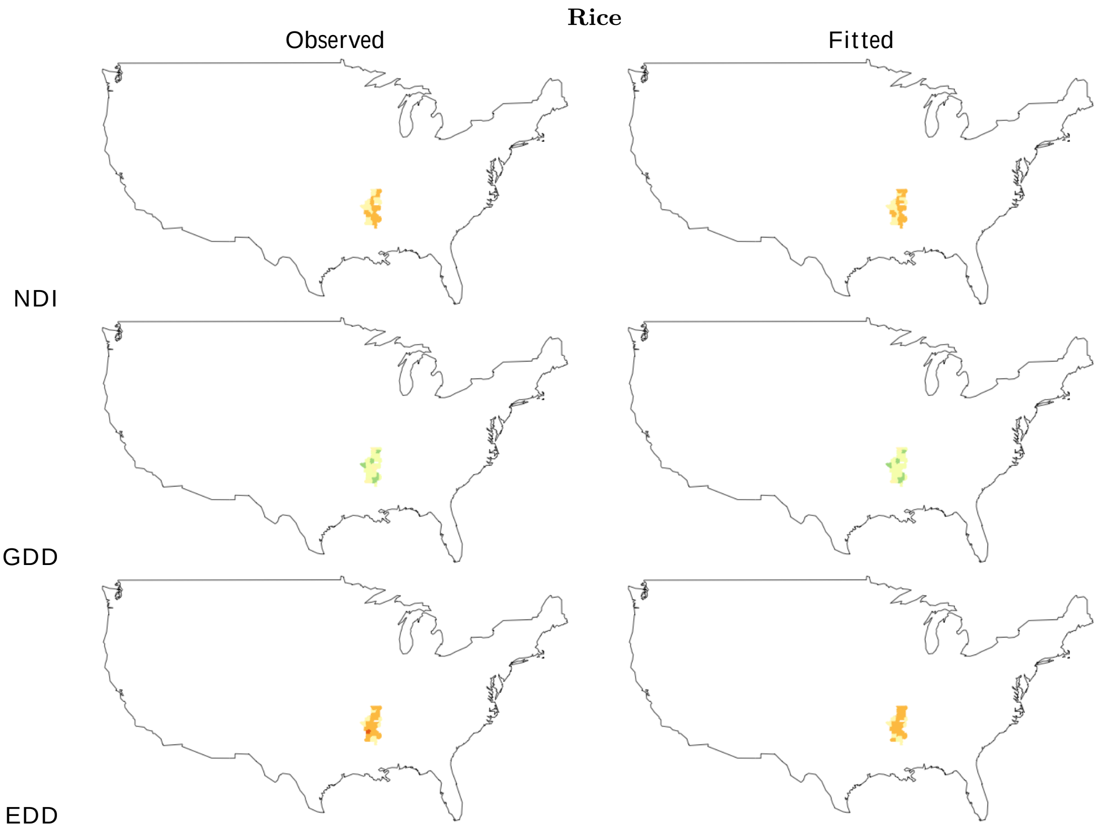

Supplementary Figure 5. Correlation coe cients between the three climate predictors and the observed Rice yield data ($R_{CDI;y}; R_{GDD;y}; R_{EDD;y}$) (left panel). Correlation coe cients between the three climate predictors and the replicated Rice yield data ($R_{CDI;y^{rep}}; R_{GDD;y^{rep}}; R_{EDD;y^{rep}}$) (right panel).

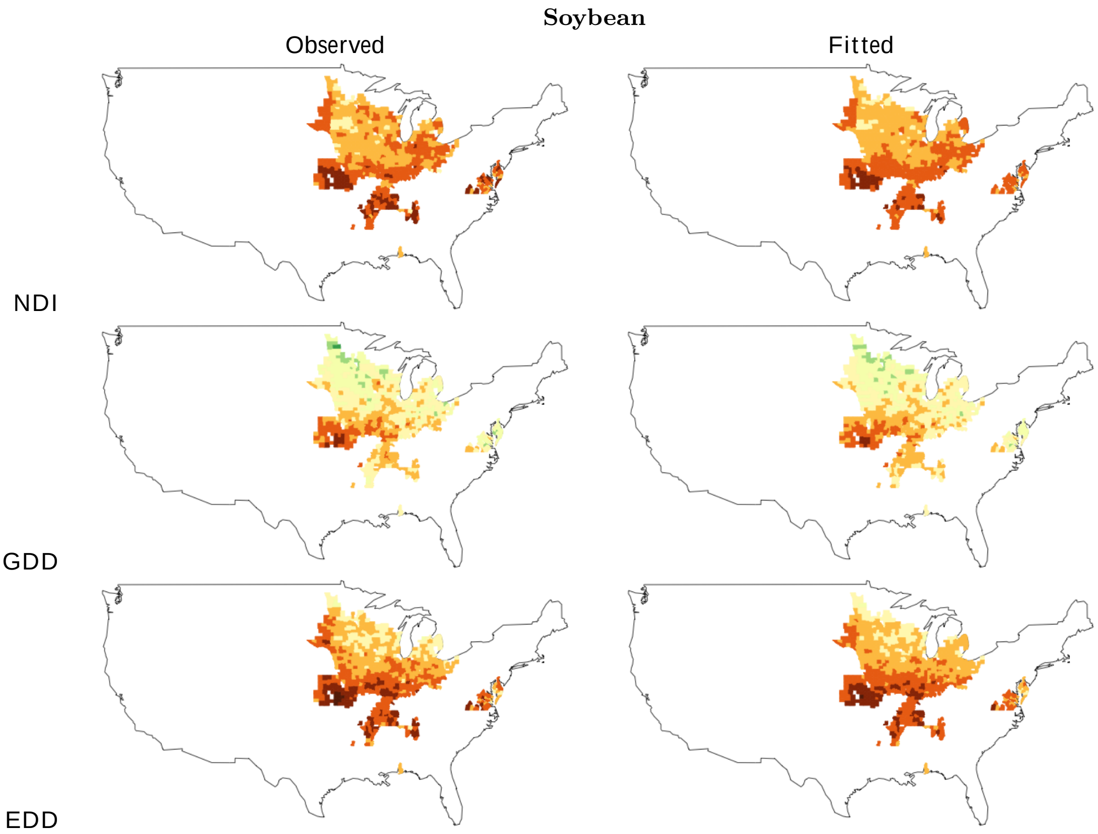

Supplementary Figure 6. Correlation coe cients between the three climate predictors and the observed Soybean yield data ($R_{CDI;y}; R_{GDD;y}; R_{EDD;y}$) (left panel). Correlation coe cients between the three climate predictors and the replicated Soybean yield data ($R_{CDI;y^{rep}}; R_{GDD;y^{rep}}; R_{EDD;y^{rep}}$) (right panel).

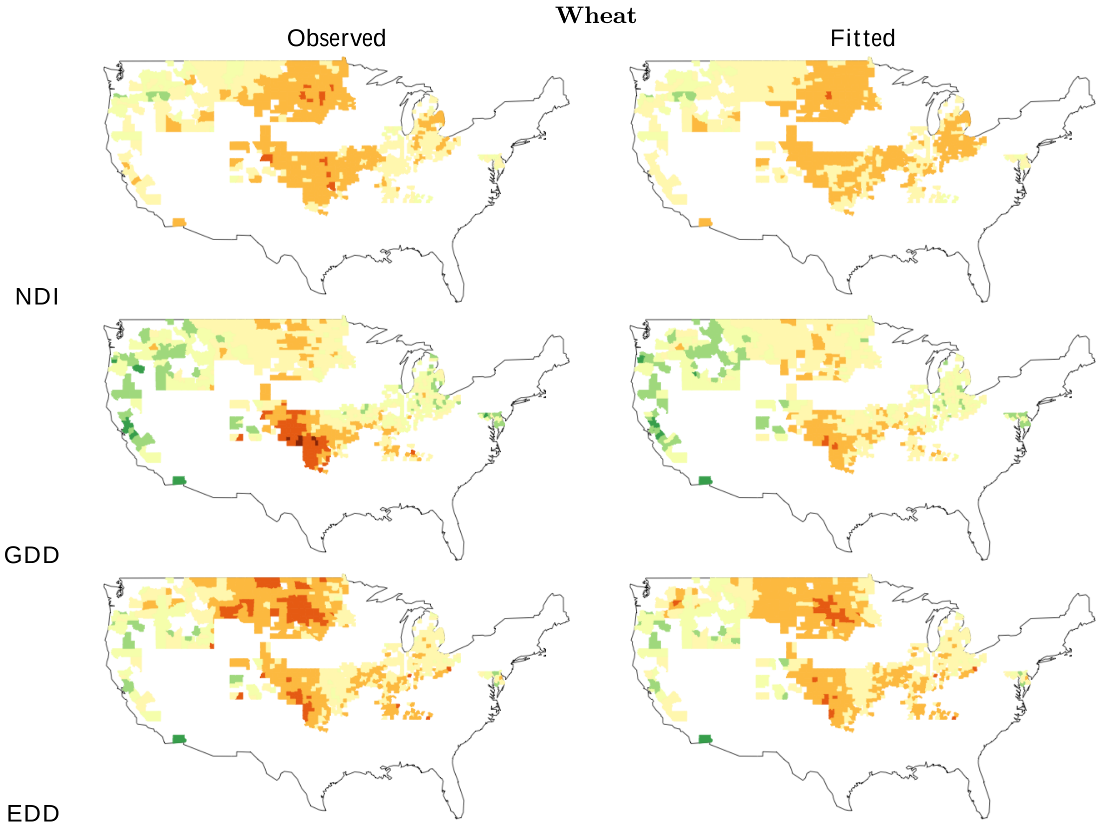

Supplementary Figure 7. Correlation coefficients between the three climate predictors and the observed Wheat yield data ($R_{CDI;y}$; $R_{GDD;y}$; $R_{EDD;y}$) (left panel). Correlation coefficients between the three climate predictors and the replicated Wheat yield data ($R_{CDI;y^{rep}}$; $R_{GDD;y^{rep}}$; $R_{EDD;y^{rep}}$) (right panel).

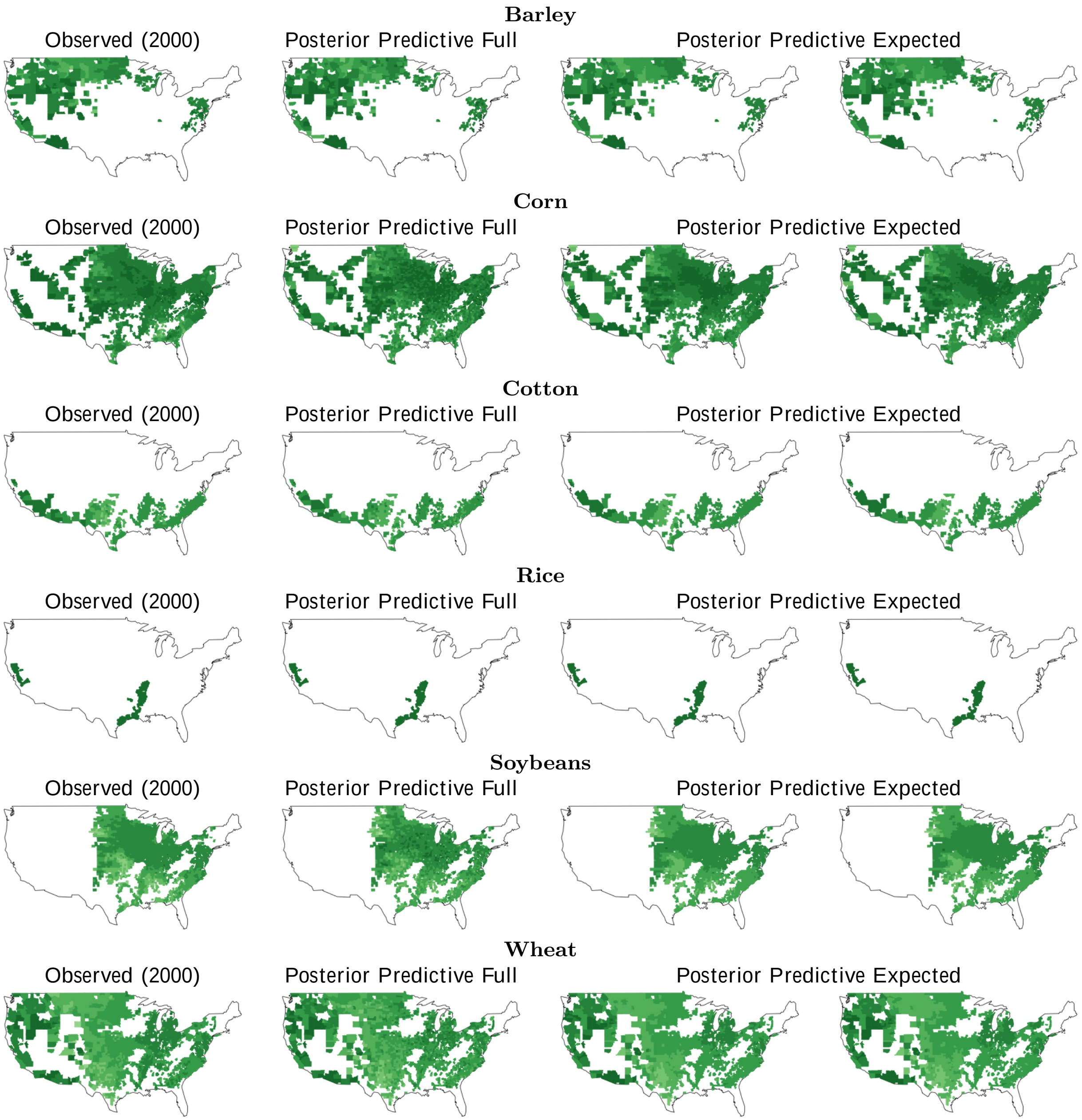

Supplementary Figure 8. Spatial posterior predictive maps. The rst column shows observed log-yields in the year 2000. The second column maps a single MCMC draw from the parameters, including the estimated error term. Since our projection model is based on expected yields, we drop the error term in the last two columns, displaying variation only coming from parameter estimates.

Supplementary Note 5: Verification of the Model Spatial Correlation

We verified the model's ability to estimate the spatial correlation in crop yields. In Supplementary Figure 9, we present this for all the crops. The scatter plot depicts the one-to-one comparison of the elements of the upper triangle of the observed correlation matrix with the expected value of the elements of the correlation matrix estimated from the posterior distribution of the yields. For an n by n full observed correlation matrix, there will be $n\frac{(n-1)}{2}$ elements in the upper triangle. These elements represent observed correlations between all the pairs of counties. Similarly, for an n by n full posterior predicted correlation matrix, there will be $n\frac{(n-1)}{2}$ elements in the upper triangle representing the predicted correlations for all these pairs. Since we have multiple such posterior predicted correlation matrices from the various simulations of the posterior predicted yields, we estimated the mean of each element across all the simulations. This mean predicted correlation matrix could now be compared with the observed correlation matrix, element-wise. The scatter plot shows this one-to-one comparison. Since not all counties cultivate these crops, the observed correlation matrices are sparse, and the comparison with the mean predicted correlation matrices could only be made based on the pairs of counties where the crops are cultivated today.

From the figure, we can see that the Bayesian model was estimating the spatial correlation in the predicted yields reasonably well, although with a slight underestimation across all crops. Note that we did not explicitly model the spatial auto-correlation in the residuals. Hence, an underestimation is expected. We assume that the modest replication of the spatial correlation in yields is a result of the pooling of the model coefficients using spatial covariates in the second level. An explicit multivariate distribution for crop yields with the covariance matrix modeled using a variogram could provide an improved representation of the spatial correlations.

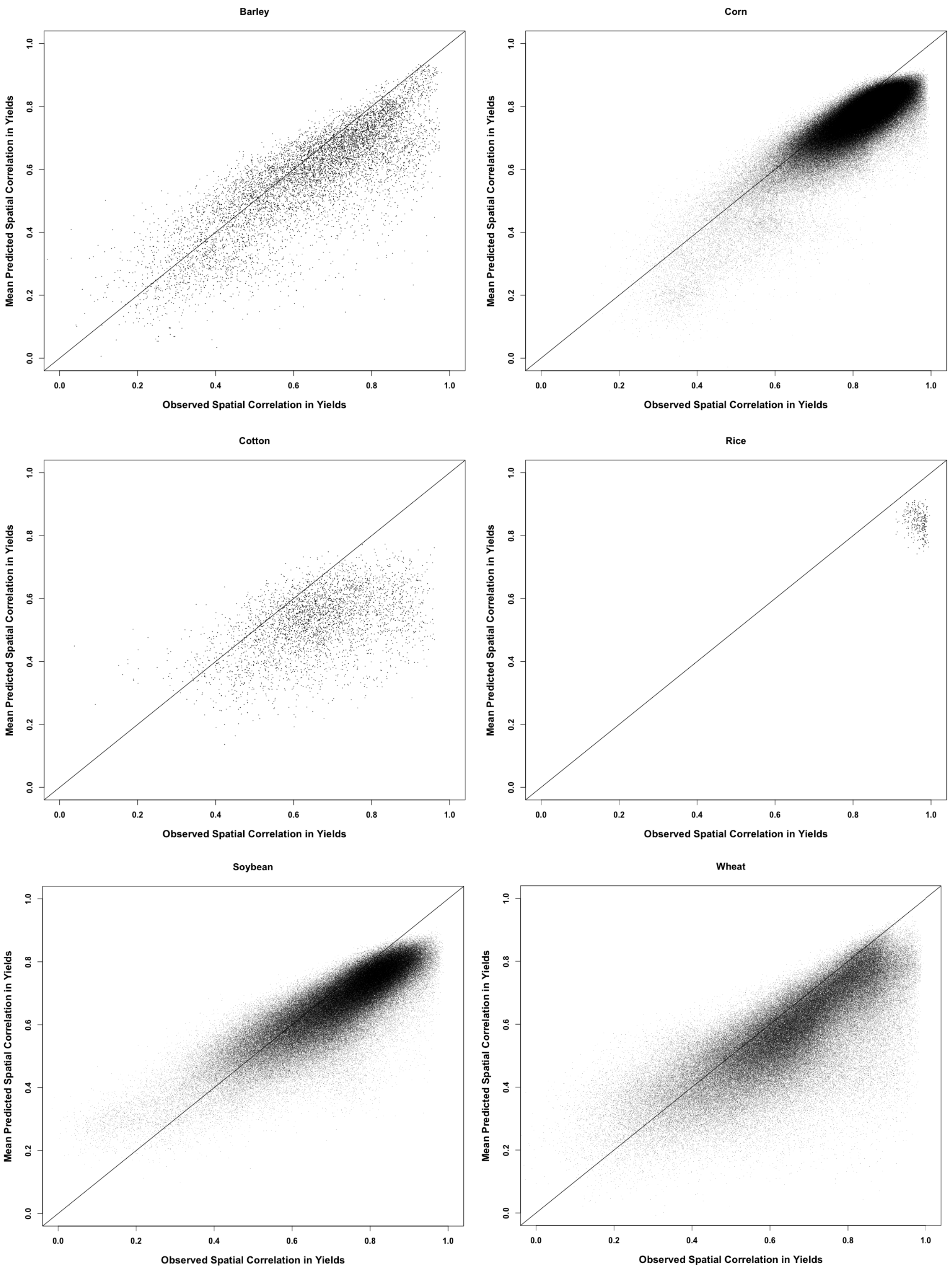

Supplementary Figure 9. Comparison of the mean predicted spatial correlation in yields with the observed correlation in yields across all the counties.

Supplementary Note 6: Model Fitted Crop Coefficient Maps and Values

The estimated coe cients for each county are shown in Supplementary Figures 10 - 15. For counties in which the given crop was not observed, we limit the coe cient on CDI (water de cits) and EDDs (extreme temperatures) to be negative. This is done by dropping posterior draws above 0.

The estimated hyper-parameters are shown in Supplementary Table 5.

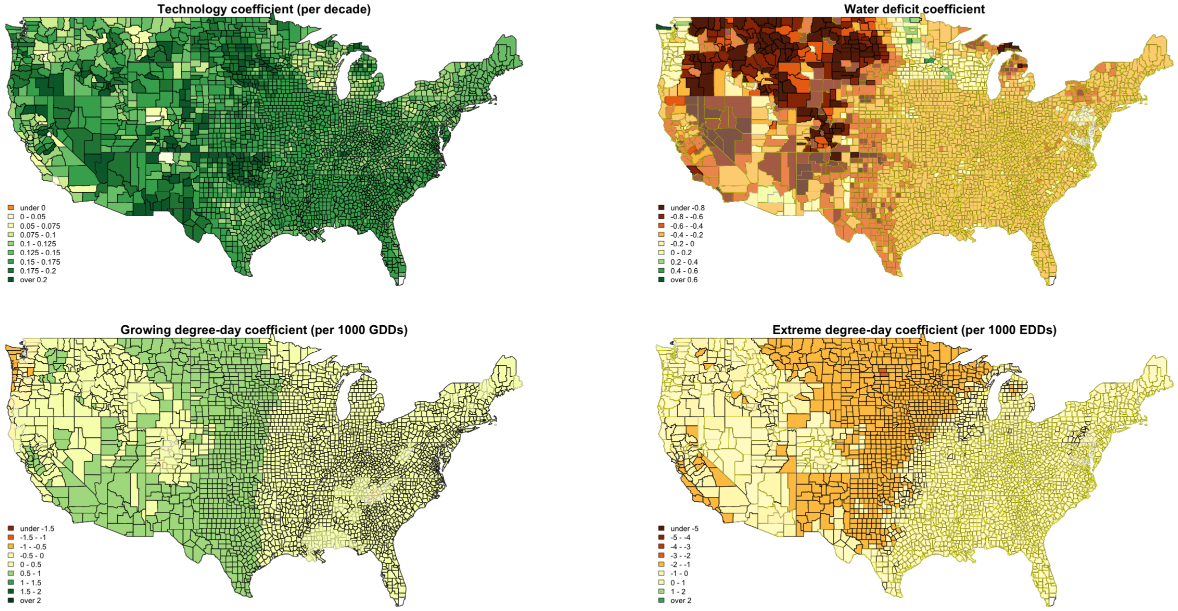

Supplementary Figure 10. Level 1 coe cients for barley. Outline color indicates the con dence level (solid black outline: >95% of posterior draws have the same sign; thin white outline: <67% of posterior draws have the same sign). **Top-left:** Technology trend. **Top-right:** Water de cit (CDI) coe cient. **Bottom-left:** GDD coe cient. **Bottom-right:** EDD coe cient. Faded counties in CDI and GDD maps have coe cients constrained to be negative.

io 12

Supplementary Figure 11. Level 1 coe cients for corn. Outline color indicates the con dence level (solid black outline: $> 95\%$ of posterior draws have the same sign; thin white outline: $< 67\%$ of posterior draws have the same sign). Top-left: Technology trend. Top-right: Water de cit (CDI) coe cient. Bottom-left: GDD coe cient. Bottom-right: EDD coe cient. Faded counties in CDI and GDD maps have coe cients constrained to be negative.

Supplementary Figure 12. Level 1 coe cients for cotton. Outline color indicates the con dence level (solid black outline: $> 95\%$ of posterior draws have the same sign; thin white outline: $< 67\%$ of posterior draws have the same sign). Top-left: Technology trend. Top-right: Water de cit (CDI) coe cient. Bottom-left: GDD coe cient. Bottom-right: EDD coe cient. Faded counties in CDI and GDD maps have coe cients constrained to be negative.
